# A national cross-sectional analysis of selenium intake and risk of osteoarthritis: NHANES 2003–2016

**DOI:** 10.3389/fpubh.2022.1047605

**Published:** 2023-01-09

**Authors:** Xiaoyu Deng, Yongqiong Tan

**Affiliations:** ^1^West China Hospital, Sichuan University, Chengdu, Sichuan, China; ^2^West China School of Nursing, Sichuan University, Chengdu, Sichuan, China

**Keywords:** dietary selenium intake, osteoarthritis, NHANES, risk factor, arthritis

## Abstract

**Background:**

The association between dietary selenium intake and arthritis, rheumatoid arthritis (RA), and osteoarthritis (OA) is inconsistent in previous studies and remain unclear. To investigate their relationship, this study was performed.

**Methods:**

Data from the National Health and Nutrition Examination Survey (2003–2016) were downloaded and further analyzed. Dietary Se intake was classified according to quartiles with quartile 1 (Q1) having the lowest intake and quartile 4 (Q4) having the highest intake. Weighted logistic regression was used to investigate the association between dietary selenium intake and arthritis, RA, and OA. Subgroup analyses were performed to verify the findings. To further examine the non-linear relationship between dietary selenium intake and OA, restricted cubic spline (RCS) was adopted.

**Results:**

In the crude model, the highest level of dietary selenium intake was siginificantly associated with decreased risks of arthritis (OR: 0.40, 95% CI: 0.37, 0.44) and rheumatoid arthritis (OR: 0.47, 95% CI: 0.40, 0.54), respectively. In the fully adjusted model, dietary selenium intake was not associated with risk of arthritis and RA (all *P* > 0.05). Conversely, the risk of OA was noted for participants with higher selenium intake (odds ratio of quartile 4 = 1.33, 95% CI = 1.07–1.65, *P* < 0.05). In the subgroup analyses, participants with diabetes had a higher risk of OA when ingested high selenium levels than those without diabetes (*P* < 0.001). The results of RCS showed that significant overall trends were found between dietary selenium intake and osteoarthritis (*P* for overall < 0.05). However, non-linear association was not detected in this association (*P* for non-linear > 0.05).

**Conclusion:**

Using data from NHANES, this study discloses that high dietary selenium intake might be associated with risk of OA. However, the generalization of conclusion needs further examination because of the limitation of dietary questionnaire survey.

## 1. Background

As the most common joint disease, osteoarthritis (OA) is prevalent globally, especially in the aged. As summarized by Sun et al. (1), the prevalence of OA is 25.03% for lumbar, 21.51% for knee, 20.46% for cervical vertebra in the middle-aged and elderly Chinese. This figure is higher in women than in men. OA is characterized by cartilage degradation, subchondral bone, and synovium lesions, leading to obvious joint pain and finally, loss of function (2). According to the report from Cross et al. (3), OA is the leading course of global disability and the years of life lived with disability increase from 10.5 million in 1990 to 17.1 million in 2010. The resultant socioeconomic burden and adverse impact for patients are heavy (4).

Joint replacement is an effective way to treat the end-stage OA with destroyed cartilage (2). However, the joint function after surgery can be poor and worse still, the lifespan of replaced joint is limited. Patients may face reoperation, consequently bringing more costs and pain. Therefore, identifying risk factors seems requisite to suppress the high incidence in the aged. Previous studies have disclosed that genetics, obesity, unhealthy diet can increase the risk of OA (5–7). Among these risk factors, the role of trace element selenium in the onset of OA is gradually noted. In China, Wang et al. (8). recruited 1,032 subjects aged ≥50 years and found that lower selenium is associated with higher risks of OA. Kurz et al. (9) also reported that diet selenium supplementation could prevent the development of mechanically induced OA, which echoed the findings from Wang et al. However, in Turkey, Yazar et al. (10) identified no association between synovial fluid and plasma selenium and OA. This insignificant finding is replicated in a dog model of OA (11). The inconsistent results may be attributed to sample size, ethnic disparity, and economic status. Therefore, more evidence with more is required to clarify the unclear association between selenium intake and OA.

To clarify the association between selenium intake and OA, we used the data from National Health and Nutrition Examination Survey (NHANES) in Americans, which is a well-designed cohort with adequate sample size (12). In addition, we also examined the non-linear relationship between selenium intake and OA, providing more evidence to determine the threshold effects of selenium.

## 2. Methods

### 2.1. Study population

A representative, non-institutionalized sample of the U.S. population has been collected on dietary habits and health condition every two years through the NHANES since 1999 as a national survey. It combines detailed in-person interviews, physical examinations, computer-based questionnaires, and laboratory tests to collect a wide range of quantitative and qualitative information (13). A detailed description of the NHANES survey methods can be found on the website (http://www.cdc.gov/nchs/nhanes/index.htm). A written informed consent was obtained from all participants before any data collection was conducted by the National Center for Health Statistics ethics review board.

Based on questionnaire information regarding arthritis and dietary nutrients intake, we used data from seven independent waves of NHANES (2003–2016). Participants were limited to those over 20 years of age, being non-pregnant, and having completed data of dietary selenium intake and arthritis outcomes.

### 2.2. Assessment of arthritis

The arthritis was assessed *via* the following question from NHANES codebook: “Has a doctor or other health professional ever told you that you had arthritis?”. Response options were “Yes” or “No.” Rheumatoid arthritis and osteoarthritis were assessed *via* the following question: “Which type of arthritis was it?”, and response options were “Rheumatoid arthritis,” “Osteoarthritis,” “Psoriatic arthritis,” “Other,” “Refused,” and “Don't know.” Percentage of psoriatic arthritis was quite low (64 cases, 0.91%), thus we did not consider the association between dietary selenium intake and psoriatic arthritis.

### 2.3. Assessment of dietary selenium intake

NHANES collected data on food intake over two non-consecutive days, with the first interview conducted in person and the second by phone. In the current analysis, we used the mean food intake over 2 days. Participants provided details of the meals they consumed in the past 24 h, and nutrients were estimated using the Food and Nutrient Database for Dietary Studies published by the USDA (14).

### 2.4. Covariates

In this study, the following variables were considered as covariates: Age (years), sex, education (above high school, high school, or less than high school), Races (Mexican American, non-Hispanic, non-Hispanic White, other Hispanic, and other races), Alcohol intake [never, former, current (Heavy, mild, and moderate)], smoking status (former, never, now), BMI, physical activity [metabolic equivalent (MET), min/week], poverty income ratio, diabetes, and cardiovascular disease (CVD). Poverty income ratio was calculated based on previous study, from <1.3 (Low), 1.3–3.5 (Median), and >3.5 (High) (15). Participants were considered to have diabetes or CVD: self-reported doctor's diagnosis of diabetes or CVD, currently taking medicine for controlling blood glucose or combating heart disease.

### 2.5. Statistical analysis

Dietary Se intakes were classified according to quartiles with quartile 1 (Q1) having the lowest intake and quartile 4 (Q4) having the highest intake. In this study, sample characteristics were presented as means and standard deviations or as percentages, and the means and standard deviations of subjects were compared using one-way ANOVA analysis or Chi-square tests. A complex multistage probability sampling process was accounted for by survey weights, sample strata, and sample clusters. A simple linear scaling of the 2-year weights created seven-wave weights (2003–2016). The association of arthritis, rheumatoid arthritis, and osteoarthritis with quartiles of Se intake was examined using logistic regression models adjusted for multivariables. The independent association between arthritis types and dietary selenium intake was examined using three models: model 1, no adjusted variable; model 2, adjusted for age, sex; model 3, adjusted for age, sex, races, education, alcohol intake, smoking status, poverty income ratio, MET, CVD, BMI and diabetes. Odds ratios (OR) and their associated 95% confidence intervals (CIs) were used to estimate the strength of the association for multivariate models.

Additional analyses were also conducted. First, selenium quartiles were transformed to continuous variables (values range from 1 to 4) and included into logistic regression model to find the potential linear trends in the association. Second, subgroup analyses were adopted stratified by age, sex, races, education, alcohol intake, smoke, poverty income ratio, CVD and diabetes. Non-linear association test was utilized to identify the modification of association between different sub population. Third, restricted cubic spline was plotted to find the dose-response trends between dietary selenium intake and arthritis risk, four knots were defined at 25, 50, 75, and 95% percentiles of dietary selenium intake, and reference value was located at the median value. The STATA version 17.0 were used to conduct all statistical analyses. A *P*-value <0.05 was considered statistically significant.

## 3. Results

As shown in Table 1, 26,620 participants with dietary selenium intake classified by quartile were included in this study. Participants in the quartile 4 group tend to be younger, male, non-Hispanic White, mild-drinker, non-smoker, obese, engaging in more physical activity, locating at highest poverty income ratio, receiving above high school education, and having no diabetes, CVD, arthritis, rheumatoid arthritis, and osteoarthritis (*P* < 0.05).

**Table 1:** The characteristics of participants (NHANES 2003–2016).

**Variables**	**Q1**	**Q2**	**Q3**	**Q4**	***P*-value**
	***N*** = **6,666**	***N*** = **6,646**	***N*** = **6,654**	***N*** = **6,654**	
Age	60.3 (16.2)	57.2 (17.0)	52.8 (17.1)	46.8 (16.3)	< 0.001
**Education**					
Above high school	3,379 (50.8%)	3,803 (57.3%)	4,110 (61.8%)	4,180 (62.8%)	< 0.001
High school	2,085 (31.3%)	1,940 (29.2%)	1,789 (26.9%)	1,886 (28.4%)	
Less than high school	1,191 (17.9%)	896 (13.5%)	748 (11.3%)	585 (8.8%)	
**Sex**					
Female	3,738 (56.1%)	2,934 (44.1%)	2,145 (32.2%)	1,140 (17.1%)	< 0.001
Male	2,928 (43.9%)	3,712 (55.9%)	4,509 (67.8%)	5,514 (82.9%)	
**Races**					
Mexican American	985 (14.8%)	1,037 (15.6%)	1,065 (16.0%)	1,168 (17.6%)	< 0.001
Non-Hispanic Black	1,586 (23.8%)	1,365 (20.5%)	1,271 (19.1%)	1,290 (19.4%)	
Non-Hispanic White	3,057 (45.9%)	3,157 (47.5%)	3,152 (47.4%)	2,985 (44.9%)	
Other Hispanic	609 (9.1%)	571 (8.6%)	573 (8.6%)	568 (8.5%)	
Other Race	429 (6.4%)	516 (7.8%)	593 (8.9%)	643 (9.7%)	
**Alcohol intake**					
Former	1,705 (27.0%)	1,488 (23.6%)	1,237 (19.6%)	1,012 (16.1%)	< 0.001
Heavy	781 (12.4%)	985 (15.7%)	1,165 (18.4%)	1,672 (26.6%)	
Mild	1,917 (30.3%)	2,087 (33.2%)	2,383 (37.7%)	2,206 (35.1%)	
Moderate	665 (10.5%)	780 (12.4%)	815 (12.9%)	852 (13.5%)	
Never	1,255 (19.8%)	953 (15.1%)	727 (11.5%)	549 (8.7%)	
**Smoking status**					
Former	1,898 (28.5%)	1,992 (30.0%)	1,980 (29.8%)	1,755 (26.4%)	< 0.001
Never	3,487 (52.4%)	3,413 (51.4%)	3,298 (49.6%)	3,302 (49.6%)	
Now	1,273 (19.1%)	1,234 (18.6%)	1,373 (20.6%)	1,595 (24.0%)	
**BMI**					
0–20	262 (4.0%)	207 (3.2%)	218 (3.3%)	242 (3.7%)	0.054
20–25	1,520 (23.2%)	1,554 (23.7%)	1,508 (22.9%)	1,600 (24.3%)	
25–30	2,321 (35.5%)	2,382 (36.4%)	2,345 (35.6%)	2,386 (36.2%)	
>30	2,437 (37.3%)	2,402 (36.7%)	2,512 (38.2%)	2,368 (35.9%)	
Physical activity (MET, min/week)	3,342.3 (5,686.2)	3,585.5 (5,730.1)	3,906.6 (6,019.2)	4,876.7 (7,011.1)	< 0.001
**The poverty index of family income**					
0–1.5	2,471 (41.0%)	2,145 (35.1%)	2,003 (32.6%)	2,043 (33.0%)	< 0.001
1.5–3.5	2,047 (33.9%)	2,094 (34.2%)	2,046 (33.3%)	1,924 (31.1%)	
>3.5	1,515 (25.1%)	1,878 (30.7%)	2,091 (34.1%)	2,217 (35.9%)	
**Diabetes**					
DM	1,709 (26.2%)	1,484 (23.1%)	1,357 (21.1%)	1,056 (16.3%)	< 0.001
IFG	297 (4.6%)	300 (4.7%)	330 (5.1%)	316 (4.9%)	
	***N*** = **6,666**	***N*** = **6,646**	***N*** = **6,654**	***N*** = **6,654**	
IGT	312 (4.8%)	293 (4.6%)	243 (3.8%)	201 (3.1%)	
No	4,197 (64.4%)	4,361 (67.7%)	4,488 (69.9%)	4,895 (75.7%)	
**CVD**					
No	5,343 (80.2%)	5,611 (84.5%)	5,818 (87.4%)	6,121 (92.0%)	< 0.001
Yes	1,322 (19.8%)	1,033 (15.5%)	836 (12.6%)	531 (8.0%)	
**Arthritis**					
No	3,894 (58.6%)	4,353 (65.6%)	4,707 (70.9%)	5,166 (77.8%)	< 0.001
Yes	2,756 (41.4%)	2,280 (34.4%)	1,928 (29.1%)	1,478 (22.2%)	
**Rheumatoid arthritis**					
No	5,214 (91.0%)	5,456 (92.4%)	5,644 (94.4%)	5,923 (95.6%)	< 0.001
Yes	514 (9.0%)	447 (7.6%)	337 (5.6%)	272 (4.4%)	
**Osteoarthritis**					
No	4,441 (90.1%)	4,803 (92.3%)	5,038 (93.2%)	5,414 (94.3%)	< 0.001
Yes	487 (9.9%)	401 (7.7%)	370 (6.8%)	327 (5.7%)	

MET, metabolic equivalent; CVD, cardiovascular disease; BMI, body mass index; IFG, impaired fasting glycaemia; IGT, impaired glucose tolerance; DM, diabetes mellitus.

Compared to the reference level (Q1), the association between dietary selenium intake and arthritis was significantly negative in the null model [OR = 0.74 (95% CI: 0.69–0.79) for Q2, OR = 0.58 (95% CI: 0.54–0.62) for Q3, OR = 0.40 (95% CI: 0.37–0.44) Table 2], and significant linear trend was found at *P* for trend < 0.001. However, this association was insignificant after adjusting for covariates with sex and age (*P* > 0.05), and the OR of quartile 4 was 1.07 (95% CI: 0.95–1.20, Table 2). Meanwhile, this association was consistent when adjusted for other covariates, and the OR of quartile 4 was 1.06 (95% CI: 0.94–1.19, Table 2).

**Table 2:** Association between dietary selenium and arthritis using logistic regression.

**Model**	**Quartiles of selenium**				***P* for trend**
	**Q1**	**Q2**	**Q3**	**Q4**	
Model 1	1.00	0.74 (0.69–0.79)	0.58 (0.54–0.62)	0.40 (0.37–0.44)	< 0.001
Model 2	1.00	0.94 (0.85–1.05)	1.00 (0.90–1.11)	1.07 (0.95–1.20)	0.173
Model 3	1.00	0.94 (0.84–1.05)	0.99 (0.88–1.10)	1.06 (0.94–1.19)	0.282

Arthritis here means the overall arthritis.

Model 1: No adjustment; Model 2: Adjusted for age and sex; Model 3: Adjusted for age, sex, races, education, alcohol intake, smoking status, income, MET, CVD, BMI, diabetes.

Similarly, the association between dietary selenium intake and rheumatoid arthritis was non-significant as comparison to quartile 1 in the full model. In the model 1, high selenium intake was associated with the decreased risk of rheumatoid arthritis [OR = 0.83 (95% CI: 0.73–0.95) for Q2, OR = 0.61 (95% CI: 0.53–0.70) for Q3, OR = 0.47 (95% CI: 0.40–0.54), Table 3], and the association was gradually increased (*P* for trend < 0.001). In the model 2 and model 3, high selenium intake was not associated with risk of rheumatoid arthritis, and the OR of quartile 4 was 0.95 (95% CI: 0.77–1.18) and 0.94 (95% CI: 0.76–1.18; Table 3).

**Table 3:** Association between dietary selenium and rheumatoid arthritis using logistic regression.

**Model**	**Quartiles of selenium**				***P* for trend**
	**Q1**	**Q2**	**Q3**	**Q4**	
Model 1	1.00	0.83 (0.73–0.95)	0.61 (0.53–0.70)	0.47 (0.40–0.54)	< 0.001
Model 2	1.00	0.91 (0.75–1.11)	0.94 (0.77–1.15)	0.95 (0.77–1.18)	0.700
Model 3	1.00	0.91 (0.75–1.10)	0.93 (0.76–1.14)	0.94 (0.76–1.18)	0.629

Model 1: No adjustment; Model 2: Adjusted for age and sex; Model 3: Adjusted for age, sex, races, education, alcohol intake, smoking status, income, MET, CVD, BMI, diabetes.

The positive association between dietary selenium intake and osteoarthritis was found in the full model (Table 4). In the model 1, dietary selenium intake was related to the decreased risk of osteoarthritis [OR = 0.76 (95% CI: 0.66–0.87) for Q2, OR = 0.67 (95% CI: 0.58–0.77) for Q3, OR = 0.55 (95% CI: 0.48–0.64), Table 4]. However, the inverse association was found in model 2 and model 3. The OR of quartile 4 was 1.33 (95% CI: 1.07–1.65) for model 2, and 1.28 (95% CI: 1.03–1.59) for model 3, respectively (Table 4).

**Table 4:** Association between dietary selenium and osteoarthritis using logistic regression.

**Model**	**Quartiles of selenium**				***P* for trend**
	**Q1**	**Q2**	**Q3**	**Q4**	
Model 1	1.00	0.76 (0.66–0.87)	0.67 (0.58–0.77)	0.55 (0.48–0.64)	< 0.001
Model 2	1.00	0.91 (0.75–1.11)	1.09 (0.89–1.33)	1.33 (1.07–1.65)	0.005
Model 3	1.00	0.88 (0.72–1.08)	1.04 (0.84–1.27)	1.28 (1.03–1.59)	0.016

Model 1: No adjustment; Model 2: Adjusted for age and sex; Model 3: Adjusted for age, sex, races, education, alcohol intake, smoking status, income, MET, CVD, BMI, diabetes.

There was no significant difference between dietary selenium intake and osteoarthritis stratified with subgroup, except for diabetes (*P* for interaction > 0.05, Table 5). In detailed, the results indicated that this association was not supported in the subgroup analysis stratified by age, sex, races, education, alcohol intake, smoking, poverty income ratio, and CVD (Table 5). Besides, participants with diabetes had a greater risk of osteoarthritis when ingested high selenium levels than those without diabetes (*P* < 0.001), though this effect size was insignificant in each group (Table 5).

**Table 5:** Subgroup analysis between dietary selenium quartiles and osteoarthritis risk.

**Model**	**Quartiles of selenium**				***P* for interaction**
	**Q1**	**Q2**	**Q3**	**Q4**	
**Age**					0.276
< 60	1.00	0.88 (0.60–1.29)	0.95 (0.66–1.38)	1.13 (0.78–1.63)	
≥60	1.00	0.85 (0.66–1.10)	0.98 (0.75–1.28)	1.28 (0.95–1.72)	
**Sex**					0.07
Female	1.00	0.70 (0.54–0.91)	0.75 (0.57–1.00)	1.18 (0.86–1.62)	
Male	1.00	1.34 (0.91–1.96)	1.32 (0.91–1.91)	1.17 (0.81–1.69)	
**Races**					0.85
Mexican American	1.00	0.43 (0.18–1.01)	0.76 (0.36–1.60)	1.00 (0.47–2.12)	
Non-Hispanic Black	1.00	1.05 (0.63–1.73)	0.81 (0.47–1.41)	1.31 (0.76–2.25)	
Non-Hispanic White	1.00	0.82 (0.62–1.07)	0.93 (0.70–1.22)	0.92 (0.68–1.24)	
Other Hispanic	1.00	0.76 (0.34–1.69)	1.23 (0.60–2.54)	1.05 (0.47–2.31)	
Other Race	1.00	2.16 (0.92–5.06)	1.13 (0.44–2.89)	1.71 (0.70–4.17)	
**Education**					0.241
Above high school	1.00	0.85 (0.68–1.08)	0.93 (0.73–1.18)	1.01 (0.79–1.29)	
High school	1.00	0.97 (0.54–1.74)	0.88 (0.47–1.66)	1.34 (0.70–2.57)	
Less than high school	1.00	0.88 (0.33–2.31)	1.00 (0.36–2.73)	0.81 (0.25–2.59)	
**Alcohol intake**					0.343
Former	1.00	0.98 (0.62–1.54)	1.09 (0.68–1.75)	1.13 (0.68–1.87)	
Heavy	1.00	0.67 (0.32–1.39)	0.58 (0.27–1.23)	1.35 (0.70–2.61)	
Mild	1.00	0.88 (0.63–1.22)	0.96 (0.69–1.32)	0.81 (0.56–1.15)	
Moderate	1.00	0.90 (0.52–1.57)	1.00 (0.57–1.78)	1.32 (0.73–2.36)	
Never	1.00	0.70 (0.39–1.24)	0.78 (0.41–1.49)	0.95 (0.48–1.87)	
**Smoke**					0.107
Former	1.00	0.84 (0.59–1.20)	0.95 (0.66–1.37)	0.85 (0.57–1.26)	
Never	1.00	0.88 (0.66–1.19)	0.88 (0.65–1.20)	1.02 (0.74–1.41)	
Now	1.00	0.91 (0.53–1.55)	1.04 (0.60–1.82)	1.48 (0.87–2.50)	
**Poverty of family income**					0.119
0–1.5	1.00	0.79 (0.53–1.17)	1.05 (0.71–1.55)	1.01 (0.67–1.54)	
1.5–3.5	1.00	0.72 (0.50–1.05)	0.75 (0.50–1.12)	1.24 (0.83–1.84)	
>3.5	1.00	1.03 (0.73–1.45)	0.97 (0.68–1.37)	0.88 (0.60–1.27)	
**CVD**					0.182
No	1.00	0.81 (0.64–1.02)	0.88 (0.70–1.11)	0.93 (0.73–1.18)	
Yes	1.00	1.16 (0.70–1.94)	1.06 (0.60–1.88)	1.78 (0.96–3.27)	
**Diabetes**					0.001
No	1.00	0.88 (0.69–1.12)	0.87 (0.68–1.12)	0.90 (0.69–1.17)	
Yes	1.00	0.82 (0.54–1.24)	1.08 (0.71–1.63)	1.53 (0.98–2.38)	

Adjusted for age, sex, races, education, alcohol intake, smoking statue, income, MET, CVD, BMI, diabetes except for subgroup variable.

**Figure 1:**
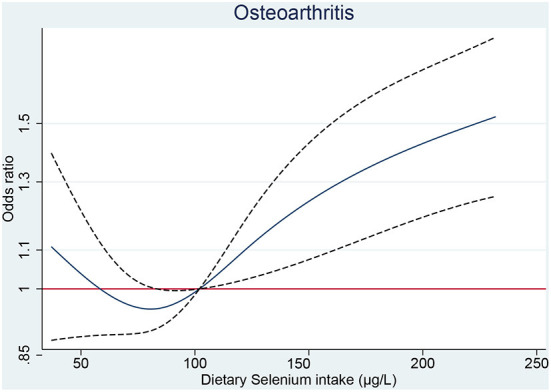
The RCS plot between dietary selenium intake and osteoarthritis. RCS, restricted cubic spline.

The restricted cubic spline showed that significant overall trends were found between dietary selenium intake and osteoarthritis (*P* for overall <0.05). However, non-linear association was not detected in this association (*P* for non-linear >0.05), which demonstrated that threshold value of dietary selenium intake for osteoarthritis needs further to research (Figure 1).

## 4. Discussion

In this study, we explored the association between selenium intake and OA in a large sample from NHANES. The risky role of high dietary selenium intake is noted for OA in Americans. Additionally, the non-linear relationship between selenium and OA is not detected, indicating the constantly rising risk with the increase of dietary selenium intake.

The association between selenium and OA is discordant in different studies. In a population-based cross-sectional study by Wang et al. (8), lower plasma selenium is found to be associated with higher risk of OA. This may be explained by the dysregulation of redox homeostasis triggered by selenium deficiency (16). The oxidative stress subsequently impairs cartilage and further leads to OA. However, as indicated by Perri et al. (17), low dietary selenium intake was common in the elderly. And the study by Wang et al. (8) was carried out among participants over 50 years old and the elderly are prone to OA, which may bias the findings. The evidence from Kurz et al. (9) in a rat model also supports this finding. They found that dietary selenium intake could prevent the progress of OA. The protective effect of high plasma selenium was also reported in a Mendelian randomization study by Qu et al. (18). But the protective effect was only observed in women, not in men, indicating the sex-specific association. In our study, high dietary selenium intake is found to increase the risk of OA, which is less reported in previous studies. Therefore, an excessive selenium intake is not recommended for the general population.

The mechanism linking selenium to OA still remains unknown. According to previous studies, metabolism associated inflammation, instead of oxidative stress, may be responsible for the adverse effect. One previous study based on mice model revealed that excessive intake of selenium could lead to the over-expression of GPx1, which further interfered with insulin signaling pathway (19). Transgenic mice overexpressing GPx1 developed hyperglycemia, and obesity, which may then trigger systemic inflammation and consequently OA (20, 21). Besides, some Se-rich food items, like red meat, processed meats, cheese and milk products has been suggested those appeared to increase risks of OA (22, 23). The high-fat and high-cholesterol food intake might raise body weight and promote OA risks.

Whether selenium supplementation is beneficial to the general population is controversial. Previous prospective studies have shown that high selenium is associated with low overall mortality (24, 25). The national survey in U.S. among 13,887 adults suggested that non-linear association was detected between increased serum selenium concentration and all-cause or cancer motility, but not in cardiovascular motility. Briefly, the inverse association was identified at selenium levels below 130 ng/ml, whereas moderate positive association was examined at selenium levels above 150 ng/ml (24). However, recent analysis from U.S. population suggested that higher serum selenium concentration could decrease the odds of all-cause and cardiovascular motility (26). In consistent results revealed that selenium intake requirement might be different across various characteristics, and corresponding threshold needs further investigation. The recommended average daily selenium intake is 60 μg for men and 53 μg for women (27). In our results (Figure 1), dietary selenium intake <100 μg don't increase the risk of OA. Hence, additional dietary selenium intake for patients with selenium deficiency are still safe and will not increase the risk of OA.

This study has some strengths and limitations. The main merit is the adequate sample size in Americans, allowing for higher statistical power. Second, sampling weights were considered in the analyses, reducing the bias from oversampling. The weighted dataset is more representative. Third, we also detected the non-linear relationship between dietary selenium intake and OA risk, providing more evidence for the threshold effect. This study also has some limitations. The principal demerit is the cross-sectional design of this study, which could not identify the causal association and avoid the bias from confounding factors. Future cohort studies should be performed to verify the findings. Additionally, the diagnosis of OA is mainly based on self-report, reducing the accuracy. Further verification using more accurate method like MRI or CT should be considered. Last but not least, we obtained dietary Se intake using questionnaire survey, which has some limitation in measurement accuracy, because of regional heterogeneity of the food production and bias from self-reported. Hence, further investigation should include biomarkers such as total serum selenium or selenoprotein P concentrations as supplement.

In conclusion, this study discloses that high dietary selenium intake is associated with risk of osteoarthritis. However, this risk is not detected for participants with dietary selenium intake <100 μg/day. For the general population, regular dietary selenium intake should be carefully considered.

## 5. Conclusions

Using data from NHANES, this study discloses that high dietary selenium intake might be associated with risk of osteoarthritis. However, current conclusion should be cautious because of limitation of questionnaire survey.

## Data availability statement

The original contributions presented in the study are included in the article/supplementary material, further inquiries can be directed to the corresponding author.

## Author contributions

XD and YT wrote, revised and reviewed the manuscript, drafted the study design, and supervised all processes. All authors contributed to the article and approved the submitted version.
